# Short‐Term Outcomes of Enhanced Recovery after Surgery (ERAS) for Ankle Fracture Patients: A Single‐Center Retrospective Cohort Study

**DOI:** 10.1111/os.13621

**Published:** 2023-01-20

**Authors:** Yuefeng Yao, Guoqing Li, Jing Li, Su Liu, Yixiao Chen, Jiapeng Deng, Yihao Wei, Liang Gao, Deli Wang, Hui Zeng

**Affiliations:** ^1^ Department of Bone & Joint Surgery Peking University Shenzhen Hospital Shenzhen China; ^2^ National & Local Joint Engineering Research Center of Orthopaedic Biomaterials Peking University Shenzhen Hospital Shenzhen China; ^3^ Renal Division, Peking University Shenzhen Hospital Peking University Beijing China; ^4^ Center for Clinical Medicine Huatuo Institute of Medical Innovation (HTIMI) Berlin Germany

**Keywords:** American Orthopedic Foot and Ankle Society (AOFAS), Ankle fracture, Enhanced Recovery After Surgery (ERAS), Medical quality and efficacy

## Abstract

**Objective:**

Enhanced recovery after surgery (ERAS) has been successfully adopted for the improvement of medical quality and efficacy in many diseases, but the effect thereof for ankle fracture patients can vary. The aim of the present study was to explore the short‐term postoperative outcomes of ERAS among ankle fracture patients.

**Methods:**

The present study was a retrospective cohort study conducted between January 2019 and May 2019. One hundred and sixty ankle fracture participations (58 males and 102 females, aged 41.71 ± 14.51 years) were included. The participants treated with open reduction and internal fixation were divided into two groups (non‐ERAS vs. ERAS) depending on whether ERAS was applied. Postoperative outcomes included American Orthopedic Foot and Ankle Society (AOFAS) score, length of stay (LOS), hospital cost, complications, and consumption of opioids. To assess the association between the groups and outcomes, generalized estimating equation (GEE) modeling and multivariable linear regression analysis were performed.

**Results:**

The average follow‐up periods of the participations were 24 months postoperatively. No significant differences were detected between the non‐ERAS group and ERAS group with respect to the demographic of patients in terms of gender, age, Danis‐Weber classification of fracture, dislocation of ankle joint, and comorbidity (*P >* 0.05). Significant differences in terms of a higher AOFAS score were found in the ERAS group compared with the non‐ERAS group (6.73, 95% CI, 5.10–8.37, *p* < 0.001) at 3 months postoperatively (PO3M) and (4.73, 95% CI, 3.02–6.45, *p* < 0.001) at 6 months postoperatively (PO6M). However, similar AOFAS scores were found at 12 months postoperatively (PO12M) (0.28, 95% CI, −0.32 to 0.89, *P >* 0.05) and at 24 months postoperatively (PO24M) (0.56, 95% CI, −0.07 to 1.19, *P >* 0.05). Additionally, the GEE analysis and group‐by‐time interaction of AOFAS score revealed that the ERAS protocol could facilitate faster recovery for ankle fracture patients, with higher PO3M and PO6M (both *P* < 0.05). At the same time, significant differences in terms of a shorter length of stay (−3.19, 95% CI, −4.33 to −2.04, *P* < 0.01) and less hospital cost (−6501.81, 95% CI, −10955.21 to −2048.42, *P* < 0.01) were found in the ERAS group compared with the non‐ERAS group.

**Conclusion:**

By reducing LOS and hospital cost, the ERAS protocol might improve the medical quality and efficacy. The present study can provide a realistic evaluation and comparison of the ERAS protocol among ankle fracture patients, and ultimately guide clinical decision making.

## Graphical abstract

Patients with the enhanced recovery after surgery (ERAS) application could obtain higher American Orthopedic Foot and Ankle Society (AOFAS) score with length of stay (LOS) or hospital cost reduction, which could help ankle fracture patients recover faster and better in the short‐term. A better short‐term prognosis would be acquired among ankle fracture patients with the ERAS protocol applied, however, all patients would obtain comparable rehabilitation 1 year and later.

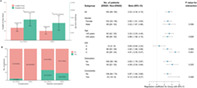

## Introduction

The ankle joint is the largest weight‐bearing joint while ankle joint trauma is the most common cause of intra‐articular fractures.^1^ Ankle fractures constitute almost 40% of sports injuries and account for approximately 10% of emergency visits.^2^, ^3^ Moreover, the amount of ankle fracture patients is steadily increasing with the development of the aging society, which often causes considerable inconvenience to daily life and increases the already large socioeconomic burden.^4^, ^5^ Meanwhile, the diagnosis‐related groups (DRGs) system has been prioritized as a payment methodology and had a significant impact on the hospital market.^6^ For inpatient healthcare, the length of stay (LOS), defined as the period between the admission date from the discharge date,^7^ is generally regarded as a crucial variable in the DRGs system.^8^ Overall, both the functional recovery and the medical quality of the ankle fracture treatment require significant improvement.

The aim of enhanced recovery after surgery (ERAS), first proposed by Wilmore and Kehlet, is to shorten patients' recovery process and improve clinical outcomes. Notably, ERAS has been successfully adopted across many surgical fields owing to the significant clinical results thereof.^9^, ^10^ ERAS is a multimodal, multidisciplinary approach based on published evidence, with the aim of reducing physical and psychological stress and achieving rapid recovery for the care of surgical patients.^11^ Previous investigations have identified various advantages among orthopedic surgical patients in that there is a tendency to obtain a higher satisfaction level with the application of the ERAS protocol compared with the traditional healthcare pathway.^12^, ^13^ However, ERAS is applied late in orthopedic trauma, and there is only one well‐developed ERAS pathway among elderly hip fractures patients.^14^ Specifically, the accelerated rehabilitation and functional recovery is critical for ensuring an augmented proper prognosis for ankle fracture patients.^15^, ^16^ Despite such importance, the effects of ERAS in terms of the short‐term postoperative outcomes have not yet been investigated thoroughly among ankle fracture patients. As such, the purpose of the present study was to: (i) compare the short‐term postoperative outcomes of ankle fracture patients with or without the application of the ERAS protocol; and (ii) explore the associations between ERAS and postoperative function, LOS, and hospital cost.

## Methods

### Ethics Statement

This retrospective cohort study was conducted in the center of the present authors from January to May 2019, and was approved by the Human Subject and Ethics Committee (2019038th) of Peking University Shenzhen Hospital, Shenzhen, China.

### Participants Selection

**Fig. 1:**
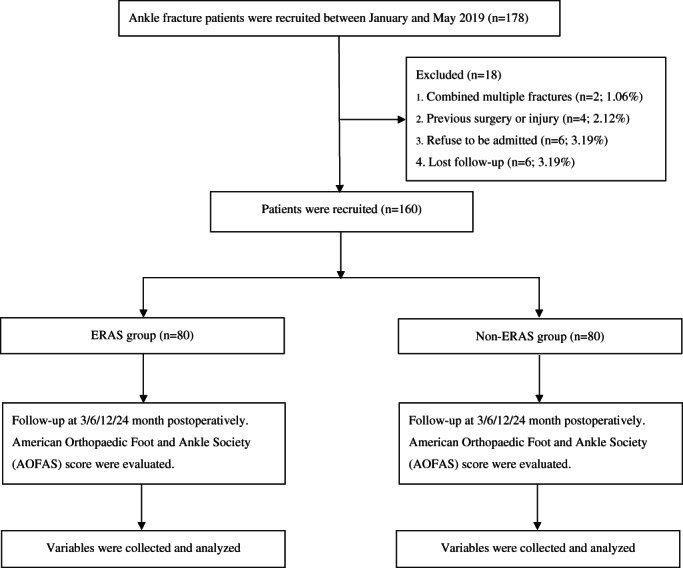
Flowchart illustrating participations selection.

The present study was a retrospective cohort study including patients who were diagnosed with ankle fractures and underwent surgery between January 2019 and May 2019 when ERAS was applied and promoted in the institution of the present authors. The exclusion criteria were as follows: (i) patients who had combined multiple fractures; (ii) patients who had undergone previous surgery or injury; (iii) patients who refused to be admitted; (iv) patients who were lost during follow‐up (Fig. 1). To ensure involvement of the routine follow‐up at 3, 6, 12, and 24 months postoperatively, qualified patients were identified from the medical record database according to the selective criteria. Moreover, the availability of relevant data including baseline parameters, demographics, radiographs, and function outcomes were extracted from medical records. The patients were then de‐labeled and divided into two groups (non‐ERAS and ERAS) according to the treatment protocols, respectively. Operations were conducted by the same experienced surgeon team specialized in ankle fracture treatments (over 500 cases annually).

### Surgical Approach

The Danis–Weber classification system was used to assist the operative planning and execution.^17^ For type A fractures (involving the lateral malleolus distal to the tibiofibular syndesmosis) and type B fractures (occurring at the level of the syndesmosis), only unstable cases were admitted for operations. For type C fractures (occurring proximal to the syndesmosis) with usually a concurrent fracture of the medial malleolus or injury to the deltoid ligament, open reduction and internal fixation were performed accordingly with an extended lateral approach or small incision for bone plate internal fixation. Open reduction and internal fixation were performed for each patient following the AO principles.^18^ To restore anatomical relationships as much as possible with the guidance of real‐time radiography, fracture reduction was intraoperatively achieved. For each patient, absolute or relative stability was restored individually according to the fracture, patient and injury requirements. Special attention was paid to preserving the blood supply to soft tissues and bones.

### The ERAS Protocol

The ERAS protocol used in the present study was established and tailored after the internal discussion among orthopedic trauma experts within the center of the present authors per the guidance of the general ERAS concept and based on the accumulative latest available evidence. Said protocol was performed in a reasonable and orderly manner according to the actual conditions of the individual patients.^19^ Such a standardized ERAS protocol, involving multi‐model analgesia, preoperative education, and minimally invasive techniques, can provide a reference for medical practitioners treating ankle fracture patients in a programed process.^20^, ^21^

**Fig. 2:**
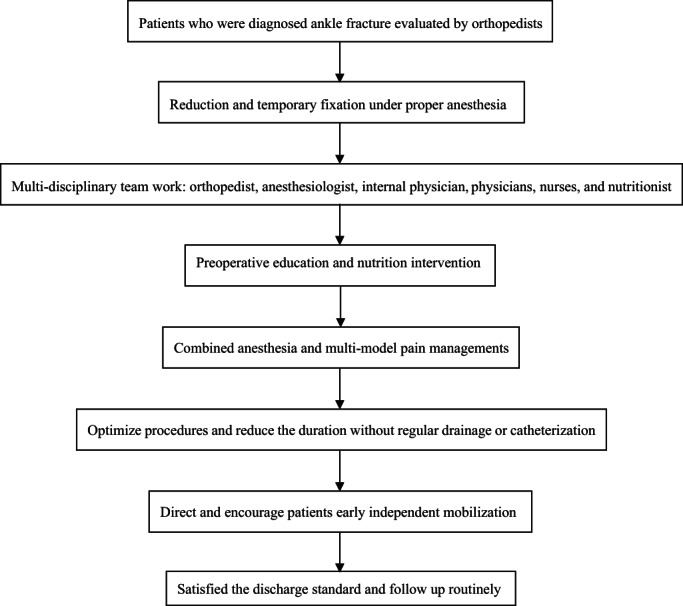
The ERAS protocol for ankle fracture patients

To reduce the risk of vascular and nerve injury, necessary reduction and temporary fixation under emergency anesthesia were performed, as well as various physical methods for detumescence during the perioperative period.^22^ Oral acetaminophen or nonsteroidal anti‐inflammatory agents were prescribed for preoperative analgesia, and oral opioids could be added if necessary. Preoperative education was conducted in a multimodal manner to reduce the depression of the patients. Nutritional screening was completed within 24 h after admission and the intervention would be performed once the nutritional risk was detected. Additionally, the recommended blood glucose control target was 7.8–10.0 mmol /L for the diabetic patients, if applicable.^23^ Nerve block combinedwith regular “background dose” of nonsteroidal anti‐inflammatory agents for postoperative analgesia and intravenous or peripheral nerve block‐controlled analgesia pump would be applied when necessary. Further, the established practical discharge criteria and routine follow‐up would be performed regularly. The described standard protocol is briefly illustrated in Fig. 2. Details about the specific ERAS protocol for ankle fracture patients in the present center were exhibited in Table 1.

**TABLE 1:** Details of ERAS protocol for ankle fracture patients in our institution

Process	Non‐ERAS	ERAS
Managements of preoperative		
MDT	Only patients with complex situations including orthopedist and specialist	Regular protocol for patients including orthopedist, anesthesiologist, nurse, and specialist
Radiograph	Ankle joint X‐ray and CT, chest X‐ray	Ankle radiograph and CT, and chest radiograph within 24 h
Laboratory test	Biological and chemistry	Biological and chemistry
Education	Inform and brochures	Inform, oral education, brochures, multimedia lectures
Psychological care	Eliminate the nervousness	Regular eliminate the adverse stress reaction and nervousness
Analgesia	Celecoxib Po 200 mg Bid	Celecoxib Po 200 mg Bid and Parecoxib 10 mg IM in the night before surgery
Feeding	Feeding was forbidden in the whole day till the operation	Protein liquid 6 h/carbohydrates liquid 4 h/ clear liquid 2h was allowed before anesthesia
Managements of intraoperative		
Anesthesia	General or combined spinal and epidural anesthesia	Combined spinal and epidural anesthesia, peripheral nerve blocks, and regular injection of cocktail around the incision
Wound drains urinary tubes	Sometimes with wound drains	No routine wound drains
	Sometimes with urinary catheterization	No routine urinary catheterization
Body temperature	Heating blanket and heat before liquid input	Monitoring, heating blanket, active warming devices, air conditioning, and heat before liquid input
Managements of postoperative		
Analgesia	Parecoxib 10 mg IM twice, celecoxib Po 200 mg Bid	Parecoxib 10 mg IM Bid for 2 days, followed with celecoxib Po 200 mg Bid for 1‐week and local cold therapy
Sleep	Estazolam if patients caught difficulty in sleeping	Regular estazolam or benzodiazepine
Nausea and vomiting	Methoclopramide if the nausea and vomiting appear	Ondansetron or seclizine for prevention
Early feeding	8 h after the general anesthesia, 6 h after the combined spinal and epidural anesthesia	Immediate supply with water and small amount of semi‐liquid food, normal feeding with high calorie/high protein/high fiber food 1 ~ 2 h postoperatively
Early mobilization	Raise the affected limb and reduce swelling, active and passive flexion and extension of the toes	Follow the directions from physical therapist, initiative, step by step, isometric and isotonic quadriceps muscle training, early out of bed without weight‐bearing activities, full range of dorsal extension and plantarflexion of toes
Follow‐up	In outpatient clinic by X‐ray	Guide the medication and exercise, observe the wound and fracture healing, evaluate the function recovery

Abbreviations: ERAS, enhanced recovery after surgery; CT, computed tomography; Bid, bis in die; IM, intramuscular; MDT, multidisciplinary team.

### Variables

The medical records were reviewed, and relevant variables were collected (by GQL and SL) retrospectively, as described in previous research.^24^, ^25^ The primary outcomes involved perioperative parameters, comorbidities, and function scores. The perioperative parameters included demographic characteristics and Danis–Weber fracture classifications.^26^ The comorbidities included hypertension, diabetes metabolism (DM), coronary heart disease (CHD), stroke, and others.^27^ The American Orthopedic Foot and Ankle Society (AOFAS) score, serving as the primary outcome (evaluated by GQL and SL), was assessed for all patients at month 3 postoperatively (PO3M), month 6 postoperatively (PO6M), month 12 postoperatively (PO12M), and month 24 postoperatively (PO24M).^28^ LOS, hospital cost, complications (including superficial incision infection, avascular necrosis, traumatic arthritis, nonunion, and malunion), and opioid consumption were secondary outcomes (evaluated by GQL and SL).

### Statistics Analysis

The distribution of data was evaluated using the Kolmogorov–Smirnov test. Normally distributed continuous variables were summarized by mean ± standard deviation (SD), while non‐normally distributed continuous variables were expressed with median (interquartile range). Categorical variables were summarized by frequency and percentage.

Participants were divided into two groups according to their treatment, and the baseline characteristics were compared between the two groups. Student *t*‐test was applied for normally distributed continuous variables while Mann–Whitney *U*‐test was performed for non‐normally distributed continuous variables. Chi‐square test was applied for nominal variables including complications and opioids consumption with the expected values being greater than five; otherwise, Fisher's exact test was performed.

To examine the association between ERAS application and AOFAS scores at each time point (month 3, 6, 12, 24 postoperatively), a generalized estimating equation (GEE) model (Gaussian family, identity link, and an autoregressive working correlation structure) was applied. The model was adjusted for gender, age, comorbidity, dislocation, and DW classification of ankle fracture. The main group effect (ERAS *vs* non‐ERAS) and the group‐by‐time interaction effect were examined.

Multivariable linear regression analysis was performed to examine the association of the group with LOS and hospital cost, which was adjusted for gender, age, comorbidities, dislocation, and DW classification of ankle fracture. In addition, multivariable logistic regression models were fitted to assess the association of the group with complications and opioids consumption, adjusted for gender, age, comorbidities, dislocation, and DW classification of ankle fracture. The results are presented as regression coefficients or odds ratios, with 95% confidence intervals (CIs).

Subgroup analyses were also displayed using a forest plot. In subgroup analysis, separate random‐coefficient models were fitted to evaluate *β* changes in participants with and without the ERAS protocol. The dependent variable was the AOFAS score at different time points postoperatively. The timepoint as the variable was entered into the models, and the coefficient thereof was regarded as the change rate of the AOFAS score, which was adjusted for gender, age (<45 years, ≥45 years), DW classification (A, B, and C), dislocation (yes / no), and comorbidity (yes / no). The analyses were stratified by whether the ERAS protocol was applied or not. Moreover, subgroup random‐coefficient models stratified by gender, age, DW classification, dislocation, and comorbidity were also performed. *P* values for group‐by‐time interaction were reported.

All tests were two‐sided, *P* < 0.05 was considered statistically significant. All statistical analyses were performed by means of R software 4.0.2 (R Foundation for Statistical Computing, Vienna, Austria).

## Results

### General Results

One hundred and sixty ankle fracture patients (58 males and 102 females, 41.71 ± 14. 51 years), undergoing open reduction and internal fixation, were grouped into the non‐ERAS group and ERAS group according to their treatment protocols. The demographic characteristics (including gender, age, fracture classification, dislocation, and comorbidities) were comparable between the non‐ERAS group and the ERAS group (*P >* 0.05) (Table 2).

**TABLE 2:** Demographic characteristics of ankle fracture patients (*n* = 160)

Variables	Non‐ERAS (*n* = 80)	ERAS (*n* = 80)	*p* value
Gender*			
Male	28 (35.00%)	30 (27.50%)	0.87^†^
Female	52 (65.00%)	50 (62.50%)	
Age (years, mean ± SD)^‡^	41.70 ± 13.96	41.85 ± 14.79	0.95
DW classification*			
A	4 (5.00%)	8 (10.00%)	0.48^§^
B	50 (62.50%)	48 (60.00%)	
C	26 (32.50%)	24 (30.00%)	
Dislocation*			
No	58 (72.50%)	48 (60.00%)	0.13^†^
Yes	22 (27.50%)	32 (40.00%)	
Comorbidity*			
No	64 (80.00%)	74 (92.50%)	0.09^§^
Hypertension	4 (5.00%)	0 (0.00%)	
DM	6 (7.50%)	4 (5.00%)	
CHD	2 (2.50%)	0 (0.00%)	
Others	4 (5.00%)	2 (2.50%)	

Abbreviations: CHD, coronary heart disease; DW, Denis–Weber; DM, diabetes metabolism; ERAS, enhanced recovery after surgery; SD, standard deviation

* The values of categorical statistics are given as the number and percentage (%) of patients.

^†^ Pearson's Chi‐squared test.

^‡^ The values of continuous statistics are given as the mean and the standard deviation.

^§^ Fisher's Exact test.

### Evaluation and Comparison of Primary Functional Outcomes

**Fig. 3:**
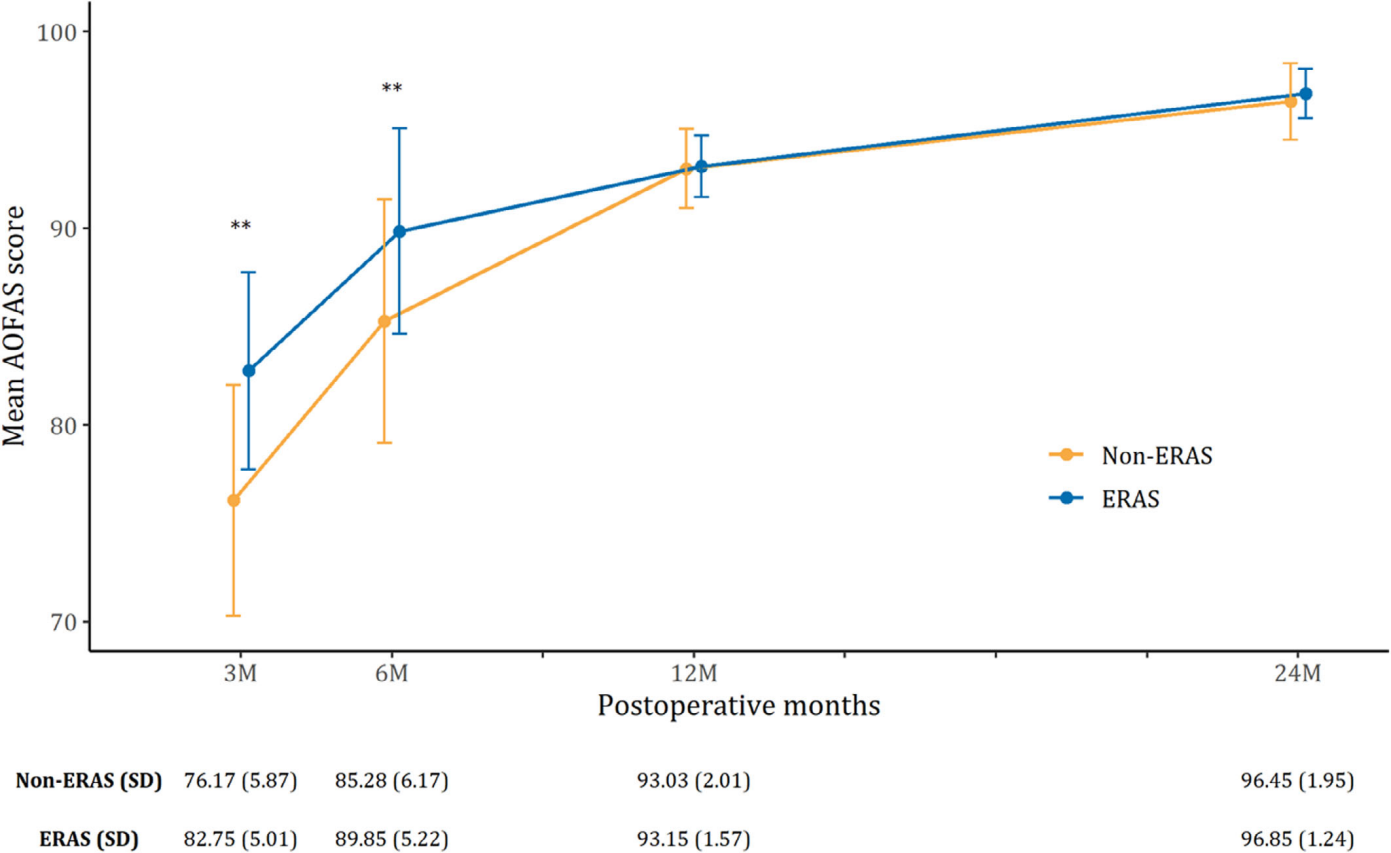
Comparison of AOFAS between the ERAS group and the non‐ERAS group (Error bar represents SD)

**Fig. 4:**
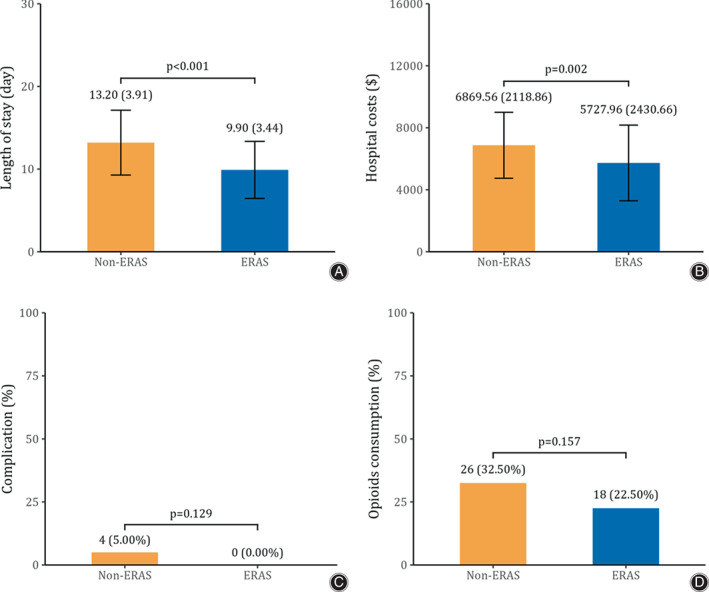
Comparison of secondary outcomes (A. length of stay; B. hospital costs; C. complication; D. opioids consumption) between the ERAS group and the non‐ERAS group (Error bar represents SD)

Higher AOFAS score could be noticed at PO3M and PO6M in the ERAS group (*P <* 0.05), but a comparable AOFAS score was observed at PO12M and PO24M (*P >* 0.05) (Fig. 3). Moreover, a shorter LOS and lower hospital costs in the ERAS group were found, as shown in the boxplots (*P <* 0.05) (Fig. 4A and Fig. 4B). Complications and opioids consumption of participants were comparable (*P >* 0.05) (Fig. 4C and Fig. 4D). GEE modeling revealed that the PO3M AOFAS score (*β* = 6.73; 95% CI, 5.10–8.37) and the PO6M AOFAS score (*β* = 4.73; 95% CI, 3.02–6.45) were higher in the ERAS group (*p* < 0.01). However, the PO12M AOFAS score (*β* = 0.28; 95% CI, −0.32 to 0.89) and PO24M AOFAS score (*β* = 0.56; 95% CI, −0.07 to 1.19) were comparable between the ERAS group and non‐ERAS group (*P >* 0.05). At the same time, group by time interaction of AOFAS score revealed that the patients in the ERAS protocol would recover better and faster, especially in terms of the AOFAS score (Table 3).

**TABLE 3:** Generalized estimating equation analysis for AOFAS score between the two groups (*n* = 160)

Time point	ERAS group *vs* Non‐ERAS group		Group‐by‐time interaction	
	Adjusted mean difference (95% CI)	*p* value	Adjusted mean difference (95% CI)	*p* value
PO3M	6.73 (5.10, 8.37)	<0.001	—	—
PO6M	4.73 (3.02, 6.45)	<0.001	−2.00 (−2.40, −1.60)	<0.01**
PO12M	0.28 (−0.32, 0.89)	0.354	−6.45 (−8.21, −4.69)	<0.01**
PO24M	0.56 (−0.07, 1.19)	0.082	−6.18 (−7.91, −4.44)	<0.01**

*Notes*: Adjusted variables: gender, age, comorbidity, dislocation, and DW classification of ankle fracture.

Abbreviations: AOFAS, American Orthopedic Foot and Ankle Society; CI, confidence interval; ERAS, enhanced recovery after surgery; PO12M, month 12 postoperatively; PO24M, month 24 postoperatively; PO3M, month 3 postoperatively; PO6M, month 6 postoperatively.

** *p <* 0.01.

### Evaluation and Comparison of Secondary Outcomes

Multivariable linear regression analysis revealed that the ERAS protocol was one of the significant contributors for reducing LOS (*β* = −3.19; 95% CI, −4.33 to −2.04) and decreasing hospital cost (*β* = −1019.09; 95% CI, −1717.11 to −321.06). Multivariable logistic regression model was applied for complications (*β* = −0.65; 95% CI, −1.42 to 0.09) and opioids consumption (*β* = −0.01; 95% CI, −0.01 to 501.31) (Table 4).

**TABLE 4:** Multivariable regression analysis of secondary outcomes between the two groups (*n* = 160)

Outcomes	Adjusted mean difference (95% CI)	*p* value
LOS (days)*	−3.19 (−4.33, −2.04)	<0.01**
Hospital cost ($)*	−6501.81 (−10955.21, −2048.42)	<0.01**
Complications^†^	−0.65 (−1.42, 0.09)	0.089
Opioids consumption^†^	−0.01 (−0.01, 501.31)	0.977

Complications: superficial incision infection, avascular necrosis, traumatic arthritis, nonunion, and malunion

Abbreviations: CI, confidence interval; ERAS, enhanced recovery after surgery; LOS, length of stay.

Adjusted variables: gender, age, comorbidities, dislocation, and DW classification of ankle fracture

* Multivariable linear regression models was applied for continuous data.

^†^ Multivariable logistic regression models was applied for category data.

^**^ *p* < 0.01.

### Evaluation the Impact of Interaction

**Fig. 5:**
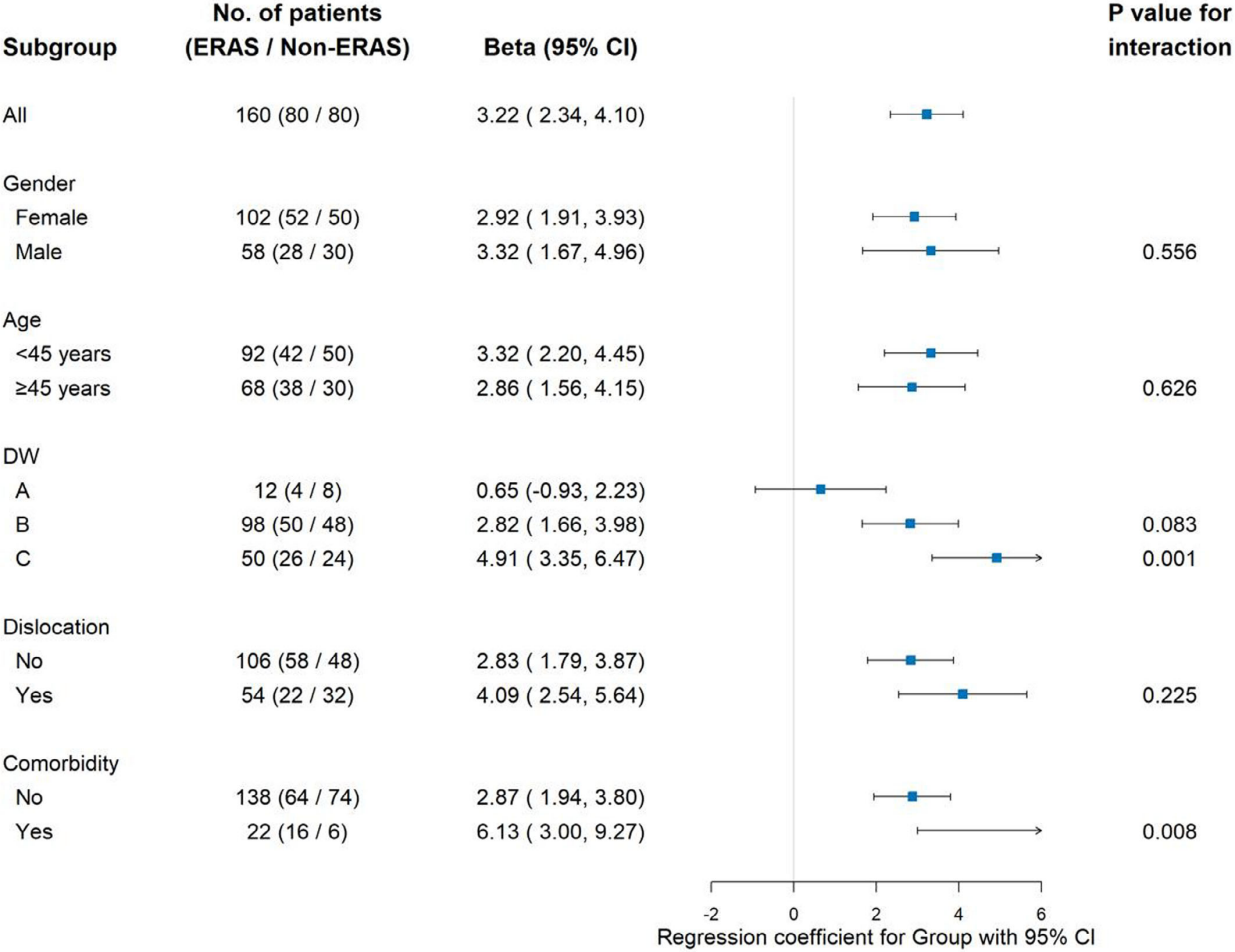
Treatments effects of interaction for the subgroup analysis (Overall and by gender, age, DW classification, dislocation, and comorbidity).

The impact of interaction is reflected in the forest plot (Fig. 5). Patients who were classified into type‐C DW classification and patients who were caught comorbidities would benefit from the ERAS protocol (*P <* 0.05).

## Discussion

The present retrospective study, in which the short‐term postoperative outcomes of the ankle fracture patients with or without the application of the ERAS protocol were compared, demonstrates that participants in the ERAS group would recover better and faster with a higher AOFAS score than that in the non‐ERAS group. Meanwhile, by reducing hospital cost and shorting the LOS, the application of the ERAS protocol might also facilitate improvement of the medical quality and efficacy.

### Better and Faster Functional Recovery in the ERAS Group Postoperatively

Ankle fracture patients in the ERAS group would obtain a better short‐term prognosis, but all patients would obtain comparable function 1 year later in both groups. The AOFAS was applied to evaluate the outcomes in addition to function recovery following ankle fractures with high reliability and validity.^29^ Consistent with previous research, the ERAS protocol could accelerate rehabilitation in terms of early‐stage within 6 months.^30^ The focus of a recent study was on rehabilitation for ankle fracture patients treated by means of a non‐ERAS protocol, with an average 27.86 ± 9.88 month follow‐up, obtaining a AOFAS score of less than 85,^31^ which was less than the recovery in the present current study. In another randomized controlled trial, ankle fracture patients received physiotherapy with an additional active controlled motion device, and a similar AOFAS score was reached.^32^ The possible mechanisms and advantages of the ERAS protocol are described as follows. First, individual conditions of participation are considered, and a patient‐center approach will optimize the management.^33^ Second, proper reduction and fixation under emergent anesthesia will allow for early mobility and pain relief.^34^ Moreover, the selection of anesthesia such as peripheral nerve blocks might also help to improve postoperative pain relief.^35^ Third, multi‐disciplinary teams (MDT) address the evaluation to deal with comorbidities.^36^ Additionally, preoperative education can comfort patients while nutrition intervention help with survival during the perioperative period, which could improve the prognosis and satisfaction of patients.^37^, ^38^

### Satisfied Clinical Outcomes in the ERAS Group

The ERAS protocol is not only a collection of evidence‐based perioperative interventions, but also an innovative MDT, which might satisfy patients by improving the medical quality and efficacy. Implementation of the ERAS protocol will benefit patients in releasing pain or stress,^39^, ^40^ reducing hospital cost,^41^ and shortening LOS,^41^, ^42^ which is in line with the results of the present study. Notably, the demographic characteristics and medical procedures might affect the clinical and economic implications.^43^ Since DRGs were mandated worldwide for curbing resource and cost‐saving implications, the application of the ERAS protocol could be a significant contributor for the implementation of DRGs.^44^, ^45^ With the application of multimodal, interdisciplinary, and interprofessional treatment concepts, the medical staff can optimize the postoperative convalescence to satisfy patients.

There were four cases in the present study with superficial incision infection caused by smoking in the non‐ERAS group, but the cases were treated with proper procedures. The ERAS protocol emphasizes the awareness of education^46^ and benefits patients who abandon unhealthy habits such as smoking. Previous publications have revealed that postoperative pain is a common issue, while opioids are effective interventions but are accomplished with undesired side effects.^47^, ^48^ Multimodal analgesia is significant in that medications are combined in different pathways,^49^ which might help to reduce consumption of opioids. Further, a recent study detailed that patients with opioid use disorders tended to experience increased odds of complications, extended hospitalization, nonhome discharge, and higher total costs.^50^ However, the development of the ERAS protocol could improve patient care and reduce cost burden, which was confirmed in the present study. Patients in the ERAS group consumed less opioids but without significant differences (*P >* 0.05), which might be due to the bias of selection or sample size from the participants. Thus, more investigation is necessary to confirm the findings of the present study. Similarly, identification of modifiable variables and components in the ERAS protocol will also benefit patients. Overall, the ERAS protocol significantly decreased the LOS and hospital cost without increasing complication rate or consumption of opioids, which adds to the evidence that the ERAS protocol could benefit and satisfy ankle fracture patients with a favorable prognosis.

### Limitations and Strengths

Several potential limitations should be addressed.^51^ First, the nature of the retrospective study might not allow for conclusive statements to be drawn while the participants were grouped non‐randomly with possible bias. Second, the significant detected association between the outcomes and the groups with the present sample as well as the significance of the ERAS protocol should be noted. Finally, the treatment model and discharge criteria might be variable. Besides the aforementioned limitations, the present study also has several strengths. First of all, quantitative published papers are relevant to ERAS, but lack details at different time points. The present investigation was the first in which the effects of the EARS protocol at PO3M, PO6M, PO12M, and PO24M were explored. In addition, all operations were performed by the same surgeon team with a standardized protocol, which made the results more reliable.

In future research, to reduce the study bias and allow the comparable appreciation of the data among different institutions, a standardized ERAS treatment process and discharge criteria might be required. Although the GEE modeling with multivariable linear regression analysis of the effects of inter‐group and group‐by‐time interaction, as well as the subgroup analyses, were performed to adjust potential confounding factors, potential biases might still be present. At the same time, the present data were only generated from a single center, which does not allow for definitive conclusions to be drawn. Therefore, future prospective multicentric randomized controlled trials with larger cohorts and long‐term follow‐ups based on large‐scale population are still required to fully understand the benefits of the ERAS protocol among the ankle fracture patients.

### Conclusions

In conclusion, better short‐term functional recovery with higher AOFAS score could be acquired among ankle fracture patients when the ERAS protocol was applied. However, all patients would exhibit comparable rehabilitation 1 year and later. Additionally, by shortening the LOS and reducing the hospital cost, ERAS might also help to improve the medical quality and efficacy. Better implementation and larger enlargement of the ERAS protocol might be necessary, allowing for further details and advantages of the ERAS protocol to be revealed for orthopedics staff.

## Funding Information

This study was supported by grants from National Natural Science Foundation of China (No. 82172432, No. 82102568, and No. 82001319), Guangdong Basic and Applied Basic Research Foundation (No. 2019A1515011290 and No. 2019A1515110983), Shenzhen Key Medical Subject (No. SZXK023), Shenzhen “San‐Ming” Project of Medicine (No. SZSM201612092), Shenzhen Research and Development Project (No. JCYJ20170307111755218, No. JCYJ20190809110807421, and No. JCYJ20210318153832004), Sustainable development project of Science and Technology in Shenzhen (No. KCXFZ20201221173411031), and Shenzhen High‐level Hospital Construction Fund.

## Author Contributions

Yuefeng Yao: Investigation, Methodology, Data curation, Formal analysis, Writing‐original draft, Writing‐review, Editing. Guoqing Li: Investigation, Methodology, Data curation, Formal analysis, Writing‐original draft, Writing‐review, Editing. Jing Li: Methodology. Su Liu: Investigation, Methodology, Data curation. Yixiao Chen: Investigation, Methodology, Data curation. Jiapeng Deng: Methodology. Yihao Wei: Methodology. Liang Gao: Investigation, Methodology, Review. Deli Wang: Investigation, Methodology, Review. Hui Zeng: Investigation, Conceptualization, Supervision, Funding acquisition, Resources, Review, Editing.

## Data Availability

The data are not publicly available due to them containing information that could compromise research participant privacy or consent but are available from the corresponding author on reasonable request with the permission of Department of bone and joint in Peking University Shenzhen Hospital.
